# CHEMICAL TESTING: Developmental Immunotoxicity in Review

**Published:** 2009-04

**Authors:** Angela Spivey

Much of the current knowledge regarding the adverse effects of environmental pollutants is based on laboratory evaluations performed using adult animals. But new clues now suggest that many diseases—including allergies, asthma, type 1 diabetes, atherosclerosis, leukemia, and autism—may originate in immune system damage caused by exposure to toxicants in early life, possibly even before birth. The routine conduct of safety screens on adult animals misses potential windows of unparalleled immune vulnerability in early life, writes immunotoxicology professor Rodney Dietert of the Cornell University College of Veterinary Medicine, in an invited review of the developmental immunotoxicity (DIT) literature published in the January 2009 issue of *Chemical Research in Toxicology*.

A central tenet of DIT is that the prenatal and perinatal immune systems are uniquely sensitive to toxic insult. For instance, prenatal exposure to dioxin causes immunotoxicity at a dose approximately 100‐fold lower than the exposure that causes damage in adults, as Dietert and colleagues reported in the January 2006 *Journal of Toxicology and Environmental Health, Part B Critical Reviews*. Moreover, different immune processes are at work in different life stages. Disease‐fighting T cells, for example, develop in the thymus during early life, but by adulthood are produced largely in other tissues. Also, some chemicals produce only transient immune system changes with adult exposure but produce lasting changes with fetal or early neonatal exposure.

Some safety screens are conducted across the life span, but DIT testing is usually initiated only after adult data suggest immunotoxicity. “Dietert’s review makes the important point that DIT testing is a complementary approach to toxicology testing in adult immune systems and should be considered when developing immune systems are likely to be exposed to xenobiotics,” says Fred Miller, chief of the NIEHS Environmental Autoimmunity Group. “However, DIT testing raises important questions regarding the appropriate doses, periods of exposure, and routes of exposure that should be tested at different phases of immune system development, all of which would depend on the expected human exposure.”

Such questions regarding how to implement DIT testing have been debated worldwide for nearly a decade. But further work is needed to refine the details of screening protocols for DIT testing, says Susan Makris, a toxicologist with the Environmental Protection Agency. Screening procedures may need to be tailored to individual chemicals. “For example, consideration must be given to the pharmacokinetics of the chemical [how the chemical is metabolized] in terms of how it’s being administered,” she says. Otherwise, testing may not closely simulate real‐world exposures.

Michael Holsapple, executive director of the Health and Environmental Sciences Institute at the International Life Sciences Institute in Washington, DC, says that what is really needed is development and implementation of DIT testing protocols, including studies to determine “triggers” that would prompt the implementation of such protocols. One such trigger might be the ability of a test compound to cross the placenta or be secreted in milk. “If the fetal or neonatal exposure seems so low as to be basically inconsequential, this could mean that DIT studies would be of little use in assessing risk,” he explains. “These and many other considerations would seem to challenge the premise that DIT testing should become a global policy.”

Once protocols are complete, regulatory change must occur across many agencies. “But even where there is agreement on what kind of testing needs to be done, implementing those studies, chemical by chemical, will be very much driven by the regulations that are enforced for each of those groups,” says Makris.

Perhaps the biggest obstacle to normalizing DIT testing is that of finding reliable ways to generate clear evidence for an impact of early‐life exposures on human health outcomes in adult life. “Discovering and characterizing the connections between chemical exposure during early‐life stages and disease onset and progression during later‐life stages will only come from developing and implementing DIT testing protocols,” says Holsapple. “And the complexity of these protocols, if they are to cover the full gamut suggested by Dr. Dietert, will be considerable indeed.”

